# Denoising Autoencoder Trained on Simulation-Derived Structures for Noise Reduction in Chromatin Scanning Transmission Electron Microscopy

**DOI:** 10.1021/acscentsci.3c00178

**Published:** 2023-06-05

**Authors:** Walter Alvarado, Vasundhara Agrawal, Wing Shun Li, Vinayak P. Dravid, Vadim Backman, Juan J. de Pablo, Andrew L. Ferguson

**Affiliations:** † Biophysical Sciences, University of Chicago, Chicago, Illinois 60637, United States; ‡ Department of Biomedical Engineering, Northwestern University, Evanston, Illinois 60208, United States; § Department of Applied Physics, Northwestern University, Evanston, Illinois 60208, United States; ∥ Department of Materials Sciences and Engineering, Northwestern University, Evanston, Illinois 60208, United States; ⊥ Pritzker School of Molecular Engineering, University of Chicago, Chicago, Illinois 60637, United States

## Abstract

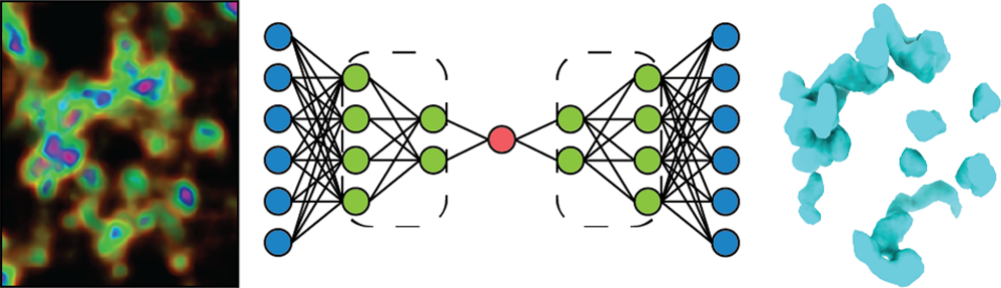

Scanning transmission electron microscopy tomography with ChromEM staining (ChromSTEM), has allowed for the three-dimensional study of genome organization. By leveraging convolutional neural networks and molecular dynamics simulations, we have developed a denoising autoencoder (DAE) capable of postprocessing experimental ChromSTEM images to provide nucleosome-level resolution. Our DAE is trained on synthetic images generated from simulations of the chromatin fiber using the 1-cylinder per nucleosome (1CPN) model of chromatin. We find that our DAE is capable of removing noise commonly found in high-angle annular dark field (HAADF) STEM experiments and is able to learn structural features driven by the physics of chromatin folding. The DAE outperforms other well-known denoising algorithms without degradation of structural features and permits the resolution of α-tetrahedron tetranucleosome motifs that induce local chromatin compaction and mediate DNA accessibility. Notably, we find no evidence for the 30 nm fiber, which has been suggested to serve as the higher-order structure of the chromatin fiber. This approach provides high-resolution STEM images that allow for the resolution of single nucleosomes and organized domains within chromatin dense regions comprising of folding motifs that modulate the accessibility of DNA to external biological machinery.

## Abstract 2

We have developed a denoising autoencoder (DAE) that outperforms other algorithms and provides high-resolution STEM images of single nucleosomes and organized domains within chromatin dense regions.

## Introduction

Chromatin is the highly organized complex of DNA, RNA, and proteins that packages DNA within the cell nucleus, prevents DNA damage, and controls replication and gene expression.^1^ The main organizational unit of chromatin is the nucleosome core particle constituting a complex of DNA wrapped around a histone octomer.^2^ Structurally, the nucleosome is approximately 146 base pairs (bps) of DNA wrapped in 1.67 left-handed superhelical turns around two copies of the H2A, H2B, H3, and H4 proteins. Chromosomes can contain hundreds of thousands of nucleosomes linked by short strands of DNA, which give it the appearance of beads on a string. The structure of these 11 nm wide nucleosomal disks is nearly conserved across all eukaryotic cells and serves as the repeating building block of chromatin.^3^ Beyond this basic structural unit, chromatin is believed to have several hierarchical levels of DNA packaging, beginning with a 10 nm fiber that further compacts into a 30 nm fiber, the latter of which has been considered to be a key intermediate level of chromatin organization and compaction within the eukaryotic nucleus.^4^ The structure of the 30 nm fiber is characterized as a nucleosomal chain folding into a solenoid or a “one-start” helical structure. Each nucleosome in this configuration interacts with its fifth and sixth surrounding nucleosomes as the nucleosomes coil around a central cavity at a rate of about six nucleosomes per turn.^5^ Though first observed under an electron microscope *in vitro*, the relevance of the 30 nm fiber *in vivo* remains an open question.^4,6,7^ More recently, studies have suggested nucleosomes can arrange themselves into stable secondary structural arrays comprised of four nucleosomes that play an important regulatory function by controlling the accessibility of DNA to external biological machinery.^8−11^ While these tetranucleosomes have been observed in reconstituted chromatin fibers *in vitro* and suggested by modeling studies *in silico*, current imaging techniques remain insufficient to resolve their existence *in situ*.^12^

Recently, chromatin staining coupled with electron and scanning transmission electron microscopy (ChromEM and ChromSTEM, respectively) have resolved the 3D organization of chromatin and observed distinct, anisotropic packing domains.^13,14^ The size and variability of these domains across different cell type have been suggested to regulate gene activity by controlling the size of macromolecular complexes that can access DNA within these clusters, thereby affecting processes such as DNA transcription, replication, and repair. In addition, variability in statistical and morphological properties of packing domains may potentially play an important role in the construction of higher-order chromatin structures such as euchromatin and heterochromatin.^15^ While these experimental imaging techniques have provided key insights into the chromatin structure, nucleosome-level packing remains obscured by statistical noise inherent to STEM imaging.^12,16^ In particular, the spatial organization of nucleosomes within dense chromatin regions suffers from low signal-to-noise ratios at these smaller length scales. Denoising STEM images provides a means to identify folding motifs and advance understanding of the details of chromatin structure, nucleosome packing, and the structure–function relation.

By combining the advances made in STEM imaging for chromatin, molecular dynamics simulations, and machine learning, we designed a deep convolutional denoising autoencoder (DAE) for STEM image denoising. Since noiseless experimental images upon which to train our denoising models are not available, we instead generate noise-free training data using by molecular dynamics (MD) simulations. This strategy is similar to the approach employed by Ziatdinov et al. in studying the surface of molecular structures.^17^ We conduct simulations of the chromatin fiber using the 1-cylinder per nucleosome (1CPN) model that has been shown to accurately reflect the possible conformations of oligonucleosomal structures.^11,18,19^ Snapshots from these MD trajectories are then converted to synthetic ChromSTEM image data sets which are used to train the DAE to remove noise artificially added to the training images and produce images with enhanced structural resolution that enable the identification and analysis of folding motifs within dense DNA regions. The DAE outperforms other well-known denoising algorithms and, as we demonstrate in applications of the trained model to experimental ChromSTEM images, resolves specific tetranucleosome motifs that induce local chromatin compaction and are known to mediate DNA accessibility. Notably, we find no evidence for the 30 nm fiber, which has been suggested to serve as the higher-order structure of the chromatin fiber.^20,21^ Our machine-learning-enabled DAE presents a means to bridge experimental ChromSTEM imaging and physics-based molecular dynamics simulations to realize high-resolution, denoised images capable of resolving previously unidentifiable tetranucleosome motifs to advance the understanding of the small-scale organization of chromatin and the relationship of structure to function.

## Methods

### Coarse-Grained Molecular Dynamics Simulations and Generation of Synthetic STEM Data

**Figure 1:**
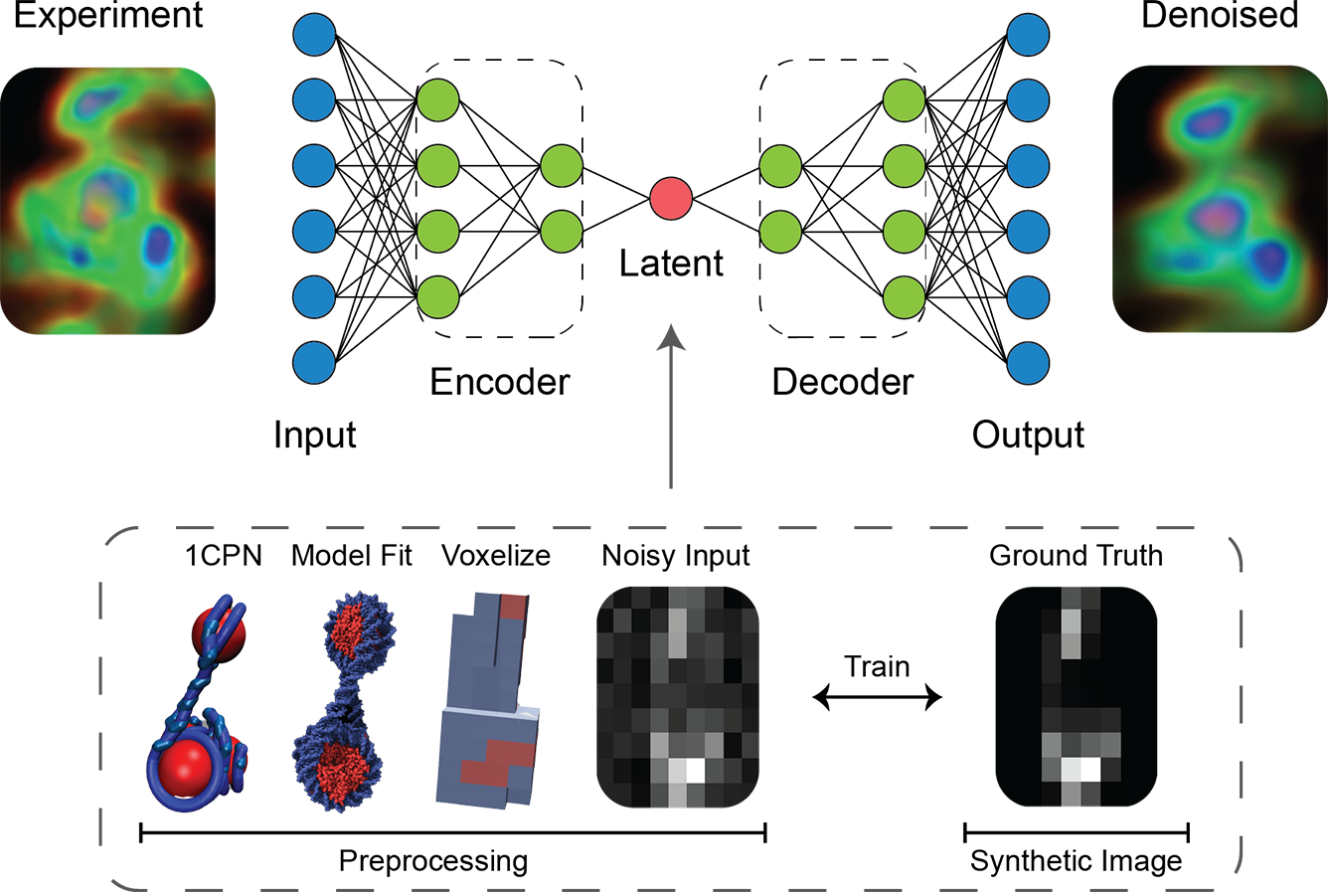
A denoising autoencoder (DAE) is constructed and trained on simulations of the chromatin fiber. We simulate nucleosome arrangements using the 1CPN model of chromatin and use the resulting trajectories to generate synthethic STEM images by superimposing crystal structures of the nucleosome (PDB: 1KX5) and DNA snippets. Noise commonly found in angle annular dark field (HAADF) STEM experiments is applied to the images and the DAE trained to remove this noise and preserve the underlying signal.

We train our DAE on tomographic images generated from MD simulations of the chromatin fiber (Figure 1). To generate a synthetic data set, coarse-grained molecular dynamics simulations were carried out using the 1-cylinder per nucleosome (1CPN) model of chromatin.^18^ The 1CPN model is parametrized by explicit experimental measurements and atomistic models of DNA that preserve molecular-level nucleosome physics enabling kilobase-scale simulations of genomic DNA. The 1CPN model is an appropriate choice, since it has been extensively validated in the literature as a reliable model for capturing chromatin dynamics.^18^ The model was fitted against experimental data and has demonstrated its ability to reproduce a wide range of chromatin processes that include nucleosome unwrapping, sedimentation coefficients, and interactions between nucleosomes, which is a primary mechanism that drives chromatin folding.^11,19^

We conducted the 1CPN simulations under conditions representative of those under which the ChromSTEM images were acquired. As anticipated, the 30 nm fiber was not observed within in our simulations, as the conditions that typically involve its formation are due to specific *in vitro* environmental conditions such as the inclusion of high-affinity 601 DNA repeats and a cationic environment (e.g., 1–2 mM Mg^2+^).^22^ Furthermore, cryo-EM images of the 30 nm fiber have not been reported for mitotic chromosomes *in vivo*.^21^ We note, however, that our pipeline is designed to be easily adaptable to new conditions and that transfer learning could be used to augment the existing model by repeating the simulations under the conditions under which the new experimental data were gathered and retraining the DAE.

After equilibration, three 30 μs replicas were conducted totaling 150 μs of simulation time of chromatin fibers varying from 150 to 200 nucleosome repeat lengths (NRLs) and comprised of 4–16 nucleosomes. The lengths and sizes were chosen to account for the natural variability in biological systems. We highlight that our simulations cover long time scales that have not been reached by previous studies. This extended simulation time allows for a more comprehensive exploration of the phase space and reduces the risk of being trapped in certain emergy minima. The 1CPN model’s effectiveness in representing chromatin behavior helps to ensure that our simulation snapshots are representative of the physical system under study. The combination of long-time-scale simulations and the use of the 1CPN model provides a strong foundation for generating a diverse and representative training data set for our denoising autoencoder. We performed an internal consistency verification that the 150 μs simulations of each system were sufficiently long to comprehensively probe the relevant configurational phase space by verifying that the phase space ensemble visited by the first 75 μs and second 75 μs produced similar distributions in key structural order parameters such as radius of gyration and root-mean-square deviation in reference to the initial elongated fiber structure.

Approximately 16000 snapshots from all simulation trajectories were extracted at 28 × 28 pixel resolution. These synthetic images represent a variety of conformations of the chromatin fiber at a resolution commensurate with that of typical ChromSTEM imaging experiments.^14,15^ From this data set, 12702 conformations were selected for training and 3176 held out as a validation set. An X-ray crystal structure of the nucleosome core particle at 1.9 Å resolution (PDB: 1KX5) was superimposed to the location of each nucleosome bead and linker DNA was built with repeating ATAT bases.^23^ Each structure was converted to a point cloud representation and then voxelized to resemble a high-angle annular dark-field scanning transmission electron microscope (HAADF-STEM) tomogram. Each synthetic image stack contained 28 × 28 × 9 voxels with a voxel dimension of approximately 3 × 3 × 3 nm^3^ corresponding to the approximate 27 nm^3^ volume captured in an experimental STEM voxel. Mathematically, the voxel intensity, *I*_*m*,*n*_, is given by the total number of atoms that are enclosed within the volume of a voxel unit, *V*_*m*,*n*_
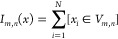
1where the position of a given atom is given by *x*_*i*_, and *m* and *n* denote the row and column indices of a voxel within a 28 × 28 × 1 voxel 2D planar slice, *I*, of the 3D voxel stack and where we have used Iverson’s bracket notation to denote the indicator function. Finally, the synthetic image intensity is normalized to match the distribution of voxel intensity in experimental tomograms.^24−26^

HAADF-STEM has emerged as a powerful imaging technique that provides nanoscale-level structural detail.^27,28^ It is, however, sensitive to environmental and instrumental noise during image acquisition that introduces extraneous signals not associated with the scattering of the sample.^16,29,30^ For example, images are acquired at different projection angles by tilting the sample stage, at high tilt angles; however, focusing becomes more difficult, which leads to image blurring.^31^ In addition, limited beam penetration and focal depth coupled with the restricted tilt range results in a lower set of projections which also introduces artifacts (i.e., “missing cone” artifacts).^32,33^ Beam damage and environmental noise (e.g., airflow, sound, temperature, etc.) also deteriorate image quality and limit the accuracy of HAADF-STEM tomographic reconstruction.^16,29,34^ Due to the particle nature of electrons and the collection method, Poisson noise remains the dominant form of noise in STEM imaging.^16,35^ To account for these effects within our simulated data, we apply several HAADF-STEM-related noise conditions including Gaussian noise, Poisson noise, and tip-blurring effects to each simulated image similar to the approach implemented by Schwenker et al.^24−26^ Parameters such as broadening effects, counts, and additive background noise were adjusted to account for the different levels of noise that may be encountered during image acquisition. Mathematically, each noise-free image, *I*, generated from the MD simulations is converted into an artificially noisy image, *Ĩ*, by corrupting it with articial noise under the noise model
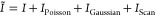
2Given that Poisson noise is not additive and correlated with voxel intensity, we instead begin by applying a signal-dependent Poisson noise layer on top of each noise-free image using the discrete probability distribution
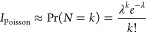
3where *N* represents the number of photons measured by a given sensor and λ is the expected number of photos per unit time interval. We make the assumption that the number of atoms counted in a given voxel unit (*I*_*m*,*n*_) is similar to photon counting in a classic Poisson process.

STEM images are susceptible to thermal vibrations and electronic noise which can be modeled as a Gaussian process.^36^ To account for this, we add a Gaussian noise layer that obeys the distribution
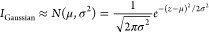
4where μ is equal to the mean of the image and σ is the standard deviation which represents the broadening (i.e., “spread”) of the signal. Similar to the approach by Schwenker et al. to emulate noise and distortion conditions common to the HAADF-STEM imaging mode, we set σ = 0.8.^24−26^

Finally, scan line shifts, *I*_Scan_, are random, persistent, time-dependent distortions that occur due to positioning errors of the electron beam that result in shifts in the image perpendicular to the scan lines.^37^ We generated this type of noise by introducing approximately a 1 subpixel offset randomly along the *x* direction and resampling these random shifts via bilinear interpolation
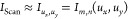
5where *u*_*x*_ and *u*_*y*_ are the desired shifts across the range [−1, 1).

### Denoising Autoencoder (DAE) Architecture

As the name suggests, denoising autoencoders (DAEs) are artificial neural networks designed to remove noise from an input signal, frequently images.^38^ A typical autoencoder is comprised of two distinct components: an encoder and decoder. The encoder compresses a high-dimensional image into a low-dimensional representation. These representations are called latent representations or encodings which the decoder uses to reconstruct the original input image. During training, the DAE is provided with training images that have been artificially corrupted with noise generated by a model representative of the noise expected to be encountered in the particular application domain. A loss function is applied that minimizes the difference between the reconstructed image and the original noise-free image. Intuitively, the training process teaches the DAE to learn a latent space representation that filters out the noise while preserving the underlying signal within the training data and permits the decoder to reconstruct denoised images.^39^ The trained DAE model may then be applied to noisy images outside of the training data for which the ground truth is unknown to predictively reconstruct denoised images. The success and generalizability of the trained model are contingent on the training images and noise model being sufficiently representative of the new images to which it is applied, and it is good practice to perform *post hoc* checks that the model has not introduced artifacts or been applied outside of its domain of applicability.

**Figure 2:**
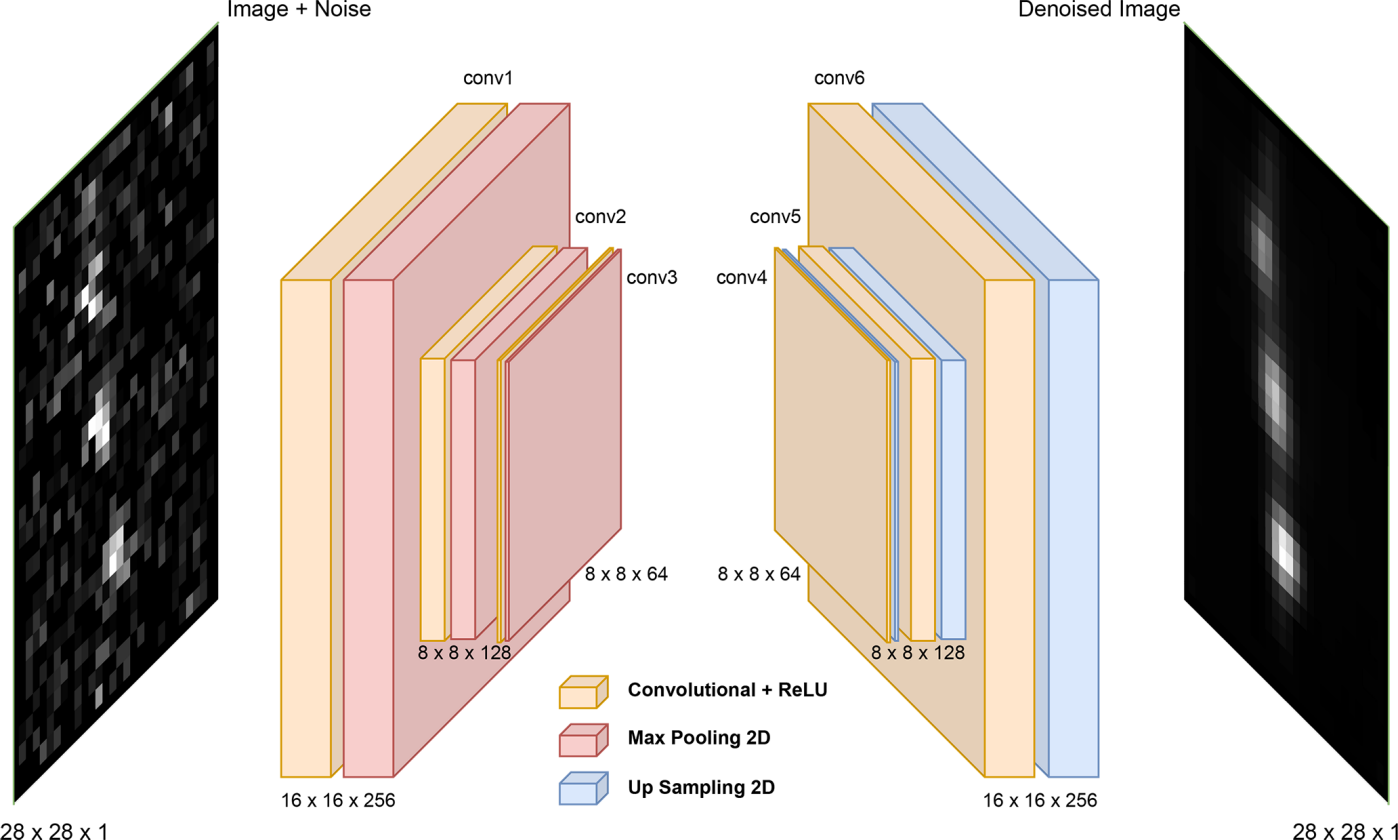
A denoising autoencoder (DAE) comprises an encoder that compresses the noisy image into a low-dimensional latent space embedding and a decoder that decompresses this embedding into a denoised image. The latent space presents an information bottleneck that the trained DAE model uses to reject noise and preserve signal, enabling reconstruction of denoised images. The DAE is trained on noise-free images for which the ground truth is known and which are artificially corrupted by noise under a noise model representative of the intended application domain for the trained DAE. The image illustrates a DAE that performs an encoding of a 28 × 28 pixel grayscale (i.e., single channel) image into a 64-channel 8 × 8 latent space embedding under three convolution plus max pooling layers, followed by decoding under three convolutional plus upsampling layers to generate a denoised 28 × 28 pixel image.^40^

We employ a fully convolutional DAE architecture that permits variable input image sizes to allow for potential variability in training and experimental image sizes.^41^ Training and validation sets of 12702 and 3176 images (80/20 random split), respectively, with 28 × 28 dimensions at a batch size of 32 were used for training and validation (Figure 2). We guard against overfitting by employing early stopping based on the validation error on a 20% randomly sampled hold-out validation partition. These images were harvested from the 1CPN MD simulations and contain a diversity of conformations of chromatin fibers at a resolution commensurate with that of a typical ChromSTEM imaging experiment. We use a convolution layer of kernel size (3,3) with 256 output filters and stride 1 employing ReLU activation functions and followed by a max pooling layer of pool size (2,2). We follow this with a second ReLU convolutional layer of kernel size (3,3), 128 output filters, and stride 1 followed by a max pooling layer of pool size (2,2), and finally a third ReLU convolutional layer of kernel size (3,3), 64 output filters, and stride 1 followed by a max pooling layer of pool size (2,2). The output of the third convolutional layer produces a low-dimensional latent space embedding of the image that serves as an information bottleneck designed to preserve the image signal and reject noise. The decoder architecture mirrors the encoder structure, employing three convolutional upsampling layers used to rebuild images to their original dimension. Our network employs a fully convolutional architecture that does not use any fully connected layers and enables its deployment on images of arbitrary size. Given that images comprise single channel grayscale pixels with intensities normalized between [0,1], the binary cross-entropy (BCE) loss function is used
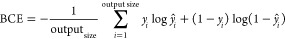
6where *ŷ*_*i*_ is the output prediction and *y*_*i*_ is the corresponding target value. It has been shown that when training autoencoders on image data, minimizing the BCE loss function facilitates gradient steps in data space from low- to high-probability regions under the data-generation distribution.^42^

We constructed and trained our DAE in TensorFlow using Keras.^43^ Training took ∼3 min per epoch on an AMD Ryzen 9 3950X 16-core CPU and Nvidia RTX 3090 GPU card. Training was performed using the Adam algorithm with a learning rate of 1 × 10^–3^.^44^ We guard against overfitting by employing early stopping based on the validation error on a 20% randomly sampled hold-out validation partition. We explored architectures employing 3–6 convolutional layers, first layer filters ranging from 2 × 2 to 5 × 5, and latent spaces bottlenecks ranging from 2 × 2 × 12 to 16 × 16 × 128 but found our result to be relatively insensitive to the precise choice of architecture. The source code for our DAE and training/validation data are available at https://github.com/Ferg-Lab/ChromSTEM-Denoising-Autoencoder.

### Denoising Performance

Denoising performance was measured using mean-square error (MSE), peak signal-to-noise ratio (PSNR), and structural similarity index (SSIM).^45,46^ Mean-square error is the total squared error between pixel intensity differences of the original noise-free image, *I*, and denoised image, *Î*, defined as
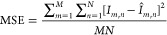
7where *M* and *N* are the number of rows and columns in the image and *M* = *N* = 28 for our training data. The lower the MSE value, the lower the error. Similarly, PSNR measures the quality of reconstruction of lossy compression by measuring the peak error and is calculated as
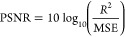
8where *R* is the maximum possible pixel value and typically depends on the bit depth of an image (e.g., for 8-bit images *R* = 255).^47^ For PSNR, the higher the value, the better the reconstruction.

Whereas MSE and PSNR calculate absolute errors between pixels, the SSIM index considers degradation as the change of perception in structural information by taking into account three key features: luminance, contrast, and structure. An SSIM value can range from −1, indicating images are structurally different, to +1, indicating they are either the same or very similar, and is defined as
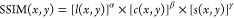
9where
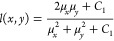
10
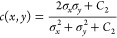
11
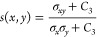
12The functions *l*(*x*,*y*), *c*(*x*,*y*), and *s*(*x*,*y*) compare luminance, contrast, and structure between two images *x* and *y*, where here we set *x* = *I* and *y* = *Î* for our ground-truth and denoised images, respectively.^47^ The variables μ_*x*_ and μ_*y*_ are their respective local means over all pixel values and represent the luminance of each images. Contrast is measured by taking the standard deviation σ_*x*_ and σ_*y*_ of all pixel values, and σ_*xy*_ is the cross-covariance of the images. The variables α, β, and γ adjust the relative importance of each feature and are typically set to unity. The constants *C*_*i*_ = (*K*_*i*_*L*)^2^ prevent functions from becoming undefined, where *L* accounts for pixel value range and is set to unity given that our images are normalized in the range of [0,1]. By convention, we adopt *C*_3_ = *C*_2_/2 and set *K*_1_ = 0.01 and *K*_2_ = 0.03.^45^

Denoising performance metrics such as MSE, PSNR, and SSIM are calculated between a ground-truth image (i.e., noise-free image), *I*, and its denoised counterpart, *Î*, produced by the DAE from the artificially noisy image *Ĩ*. Given that noise-free ChromSTEM images do not exist to serve as a ground-truth comparison, we rely on power spectral density (PSD) plots to compare raw and denoised experimental image sets. PSD represents the total signal power contributed across the frequency domain of a signal. For images, it measures the strength of the features at different resolutions. This allows for comparison of morphological features and noise in the low- and high-wavelength domains, respectively. We compute the PSD by taking the discrete Fourier transform (DFT) of each image which allows for the decomposition of resolutions
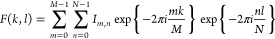
13where *I*_*m*,*n*_ is a representation of the image in the spatial domain corresponding to the grayscale intensity of the pixel at row (*m*) and column (*n*) coordinates, *F*(*k*, *l*) is the representation of the image in the Fourier domain corresponding to the Fourier component at discrete row-wise and column-wise “frequencies” *k*/*M* and *l*/*N*, and *k* = 0, ..., (*M* – 1) and *l* = 0, ..., (*N* – 1).^48,49^ Since we only consider square images for which *M* = *N*, we simplify this expression to equalize the row and column frequency components by setting *k* = *l* so that
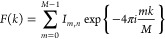
14The PSD follows from the modulus of the DFT as *P*(*k*) = |*F*(*k*)|.

### ChromSTEM Sample Preparation, Imaging, and Reconstruction for A549 Cell Nucleus

Adenocarcinoma human lung epithelial cell line A549 (ATCC Manassas, VA) was cultured in Dulbecco’s Modified Eagle Medium (ThermoFisher Scientific, Waltham, MA, #11965092) and maintained at 5% CO_2_ and 37 °C. All culture media were supplemented with 10% fetal bovine serum (Thermo Fisher Scientific, Waltham, MA; #16000044) and penicillin–streptomycin (100 μg/mL; Thermo Fisher Scientific, Waltham, MA; #15140122). The cell line was tested for mycoplasma contamination with Hoechst 33342. Cells were seeded on 35 mm glass-bottom Petri dishes (MatTek Corp.) until approximately 40–50% confluent and were given at least 24 h to adhere to the dish before fixation.

For ChromSTEM sample preparation, the previously published protocol was adapted.^13^ A549 cells cultured on the glass-bottom dishes were thoroughly rinsed three times in Hank’s balanced salt solution without calcium and magnesium (EMS). A fixation solution (2.5% EM grade glutaraldehyde, 2% paraformaldehyde, 2 mM CaCl_2_ in 0.1 M sodium cacodylate buffer, pH = 7.4) was prepared. Cells were then fixed at room temperature for 5 min and then replaced with fresh fixative and fixed on ice for 1 h. All the succeeding steps, unless mentioned otherwise, were performed on ice. After fixation, the cells were then washed with 0.1 M sodium cacodylate buffer five times on the ice. The samples were incubated in a blocking buffer (10 mM glycine, 10 mM potassium cyanide in 0.1 M sodium cacodylate buffer, pH = 7.4) for 15 min. Next, the samples were stained with 10 μM DRAQ5 (Thermo Fisher) and 0.1% saponin solution in 0.1 M sodium cacodylate buffer, pH = 7.4 for 10 min. The cells were washed with a blocking buffer twice, and then incubated in the blocking buffer on ice before photobleaching. The blocking buffer was replaced with 2.5 mM of 3–5′-diaminobenzidine (DAB) solution (Sigma-Aldrich) in 0.1 M sodium cacodylate buffer, pH = 7.4, during photobleaching which was performed on a cold stage developed in-house from a wet chamber and equipped with humidity and temperature control.

A continuous epi-fluorescence illumination (150 W xenon lamp) with a Cy5 red tilter with a 100× objective was used to bleach a spot—a random field of view with several cells—on the dish for 7 min on the cold stage. After photobleaching, the cells were washed five times with 0.1 M sodium cacodylate buffer. Reduced osmium solution (EMS) containing 2% osmium tetroxide, 1.5% potassium ferrocyanide, and 2 mM CaCl_2_ in 0.15 M sodium cacodylate buffer, pH = 7.4, was then used to stain the cells for 30 min on ice. The cells were then washed five times with double-distilled water on ice. Next, serial ethanol dehydration (30%, 50%, 70%, 85%, 95%, 100% twice) was performed on ice, and the last 100% ethanol wash was performed at room temperature. Durcupan resin (EMS) was used for infiltration and embedding. Resin mixture 1 was prepared by mixing (i) 10 mL of Durcupan ACM single-component A, M, epoxy resin, (ii) 10 mL Durcupan ACM single component B, hardener 964, and (iii) 0.15 mL of Durcupan ACM single component D. A 1:1 infiltration mixture containing equal proportions of 100% ethanol and Durcupan resin mixture 1 was used to infiltrate cells for 30 min at room temperature. Next, a 2:1 infiltration mixture containing 5 mL of 100% ethanol and 10 mL of Durcupan resin mixture 1 was used to infiltrate the cells for 2 h at room temperature. Durcupan resin mixture 1 was used to infiltrate the cells at room temperature for 1 h. Resin mixture 2 was prepared by adding 0.2 mL of Durcupan ACM, single component C, accelerator 960 to mixture 1 (10 mL of component A, 10 mL of component B, and 0.15 mL of component D). Durcupan resin mixture 2 was used to infiltrate the cells at 50 °C in a drying oven for 1 h.

The cells were embedded flat with fresh Durcupan resin mixture 2 in BEEM capsules and cured at 60 °C in a drying oven for 48 h. An ultramicrotome (UC7, Leica) was used to prepare 100 nm thick sections that were deposited onto a copper slot grid with carbon/Formvar film. Then, 10 nm colloidal gold fiducial markers were deposited on both sides of the sample. A 200 kV cFEG STEM (HD2300, HITACHI) with HAADF mode was used to collect all images. While keeping the field of view constant, the sample was tilted from −60 to 60° with 2° increments on two roughly perpendicular axes, with a pixel dwell time of ∼5 μs during image acquisition. Each tilt series was aligned with fiducial markers in IMOD and reconstructed using Tomopy with a penalized maximum likelihood for 40 iterations independently.^50,51^ The final tomogram was a 3D image size of 1230 × 1230 × 100 nm with a nominal voxel size of 2.9 nm.

## Results and Discussion

**Figure 3:**
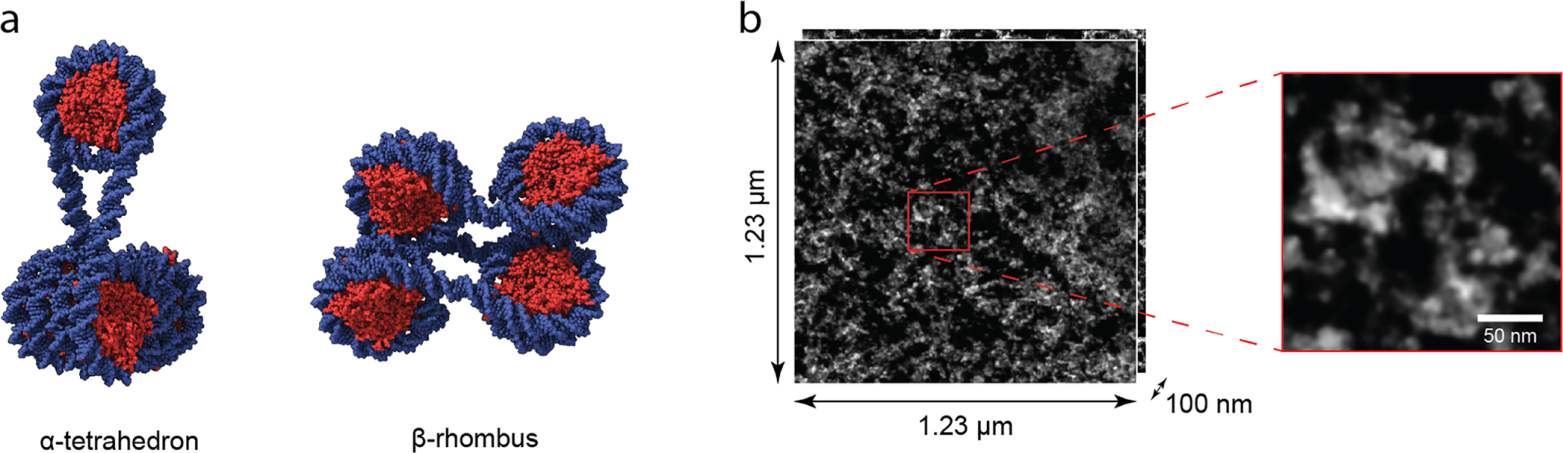
Resolution in dense chromatin regions is obstructed by the intrinsic noise of STEM imaging. (a) The α-tetrahedron and β-rhombus tetranucleosome motifs have been proposed to play a regulatory and epigenetic role in the accessibility of DNA to external cellular machinery. The α-tetrahedron promotes DNA compaction, whereas the β-rhombus results in elongated chomatic structures. Histone proteins are colored in red, and DNA is colored in blue. (b) In this work we employ high-resolution ChromSTEM tomograms comprised of 33 slices at 1.23 μm × 1.23 μm × 100 nm. The structural resolution accessible to experimental ChromSTEM tomograms is limited by the conformational variability of chromatin within chromatin-rich regions, Poisson noise, and the ability of image segmentation approaches to differentiate background and chromatin signal by voxel intensity.

Tetranucleosomes are widely considered the building block of the chromatin fiber and have been crystallized and observed in cryo-EM images of longer chromatin fibers.^9^ Recent studies have suggested the existence of two tetranucleosome motifs that regulate gene expression—the α-tetrahedron and β-rhombus (Figure 3a).^10,11^ Experiments and modeling studies have indicated that these two energetically stable conformations may induce local chromatin compaction (α-tetrahedron) or the formation of elongated aggregates (β-rhombus) and are therefore proposed to play important regulatory and epigenetic roles in the accessibility of DNA to external machinery such as transcription factors.^10,11,52,53^ While ChromSTEM has been able to resolve variably packed nucleosomes and linker DNA segments at ∼2 nm spatial resolution, the variation of size, density, and shape of chromatin rich regions can obstruct finer-scale resolution of the structural arrangement of nucleosomes (Figure 3b). The structural resolution is also degraded by Poisson (i.e., shot) noise associated with electron counting statistics and the relatively poorer performance of segmentation (i.e., differentiation of background and chromatin signal by voxel intensity) within chromatin-rich regions relative to regions where nucleosomes are well-separated and have uniform intensity.^16^ We develop a machine-learning-assisted computational denoising platform by training a denoising autoencoder (DAE) over coarse-grained molecular dynamics simulations and apply the DAE to *in situ* high-resolution HAADF ChromSTEM microscopy images of chromatin within mammalian cell lines to resolve tetranucleosome motifs.

### Testing on Synthetic Data

**Figure 4:**
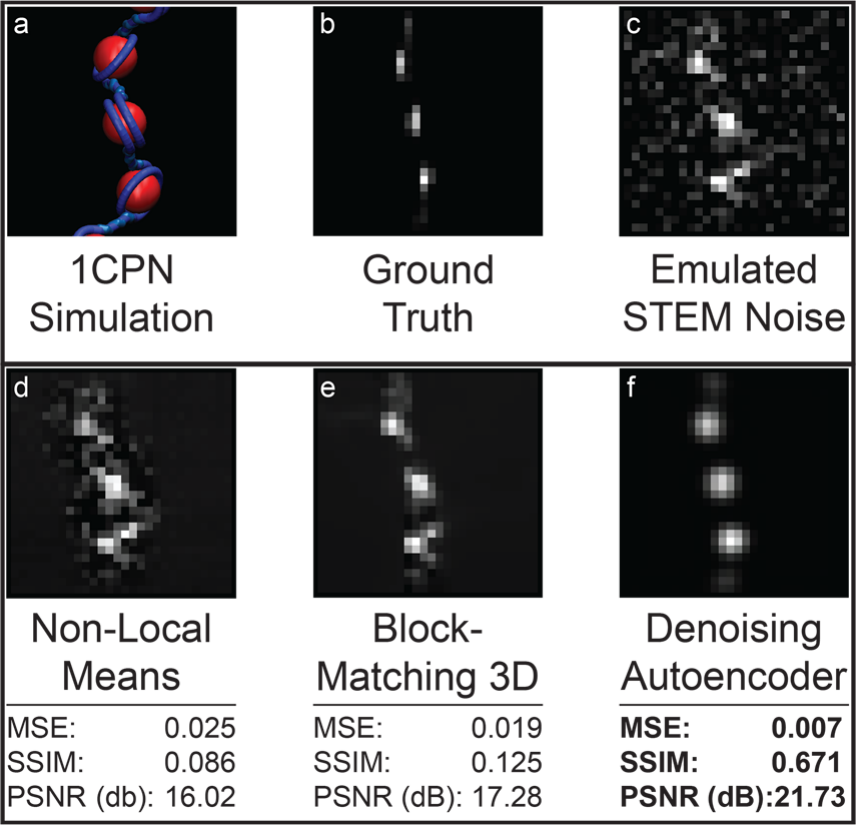
Illustrative example of DAE denoising performance to one selected synthetic ChromSTEM test image harvested from the 1CPN MD simulations. (a) The selected snapshot was harvested from 1CPN MD simulations of chromatin fibers varying from 150 to 200 nucleosome repeat lengths (NRLs) comprised of 4–16 nucleosomes. (b) The noise-free synthetic ChromSTEM image *I* was constructed from the MD snapshot using eq 1. This constitutes the ground truth image against which we evaluate denoising performance. (c) The noisy image *Ĩ* was generated by adding artificial noise representative of that found in angle annular dark field (HAADF) STEM experiments to the noise-free image using eq 2. The denoised image *Î* produced from the noisy test image by (d) nonlocal means (NLM), (e) block-matching and 3D filtering (BM3D), and (f) the DAE. The DAE outperforms NLM and BM3D along all three performance metrics (low MSE, high PSNR, high SSIM) for this particular image and over all 3000 test images (cf. Table 1).

To validate our trained DAE, we first tested its performance against standard denoising techniques in an application to synthetic ChromSTEM images to which artificial noise was added and the ground truth (i.e., noise-free) images were exactly known. We collected 3000 test images harvested from 1CPN MD simulations of chromatin fibers varying from 150 to 200 nucleosome repeat lengths (NRLs) and comprised of 4–16 nucleosomes and converted these into noise-free images *I* and noisy images *Ĩ* using eqs 1 and 2. Importantly, the test set data were never exposed to the DAE at any point during their training. We report in Table 1 the denoising performance of our DAE compared to the popular nonlocal means (NLM) and block-matching and 3D filtering (BM3D) techniques.^54,55^ Performance is assessed using the mean square error (MSE), structural similarity index (SSIM), and peak signal-to-noise ratio (PSNR) metrics that are commonly used to benchmark denoising methods.^46^ Better performance is associated with a reduction in cumulative squared error between the compressed and the original image (lower MSE), an increase in the ratio between the maximum possible power of an image and the power of corrupting noise (higher PSNR), and preservation of structural information between the reference and denoised image (higher SSIM). We present in Figure 4 an illustrative example of the application of each of the three denoising approaches to a representative snapshot taken from the 3000 test images.

**Table 1:** Mean and Standard Deviation for 3000 Synthetic ChromSTEM Test Images Calculated to Compare the Denoising Performance of Our DAE against Nonlocal Means (NLM) and Block-Matching and 3D Filtering (BM3D)^a^

denoiser	MSE	SSIM	PSNR (dB)
NLM	0.011 ± 0.003	0.15 ± 0.04	20 ± 1
BM3D	0.007 ± 0.004	0.55 ± 0.17	22 ± 2
**DAE**	**0.003** ± **0.001**	**0.83** ± **0.04**	**26** ± **2**

a Snapshots were harvested from 1CPN MD simulations of chromatin fibers varying from 150 to 200 nucleosome repeat lengths (NRLs) and comprised of 4–16 nucleosomes and converted into noise-free images *I* and noisy images *Ĩ* using eqs 1 and 2. Denoising performance is compared using the mean square error (MSE), structural similarity index (SSIM), and peak signal-to-noise ratio (PSNR) metrics. The DAE outperforms nonlocal means and BM3D along all three performance metrics (low MSE, high PSNR, high SSIM).

Our DAE performed the best in all three denoising performance metrics (MSE = 0.003, SSIM = 0.83, PSNR = 26 dB), followed by BM3D (MSE = 0.007, SSIM = 0.55, PSNR = 22 dB) and nonlocal means (MSE = 0.011, SSIM = 0.15, PSNR = 20 dB). This represents a 57% improvement in MSE relative to BM3D and 72% improvement over nonlocal means (Table 1). From the example in Figure 4, we can see that our denoising autoencoder is not only able to remove the applied Gaussian and Poisson noise but also has the ability to account for distortions which are typical to STEM experiments by virtue of the fact that it was trained on 1CPN molecular dynamics training data that preserve the physically representative structure of the chromatin strand. Given that denoising autoencoders are inherently lossy compression methods, some fuzzy imaging or loss of information is expected during the encoding process which can lead to broader output signals. The primary goal of our DAE method is to achieve a balance between noise reduction and preservation of structural features in the ChromSTEM images. While it might be possible to reduce these broader signals further, doing so could compromise the performance of the DAE or lead to overfitting.

We do observe that although our test does expose the DAE to novel synthetic ChromSTEM images it has not before encountered, they are generated using the same model as the training data. Conversely, the nonlocal means and BM3D approaches are standard algorithms that are not trained over images from a particular domain and are more general-purpose denoising tools. As expected, the DAE appears to have learned to distinguish the physical arrangement of nucleosomes along the chromatin fiber within the physics-based simulation training data from the applied noise model and can use these learned patterns to effectively denoise new synthetic ChromSTEM images that it has not previously encountered. A possible cost of this learning is, of course, that the DAE will likely not serve as a good general-purpose, application-agnostic denoising algorithm in the same manner as nonlocal means and BM3D.

### Application to Experimental Data

**Figure 5:**
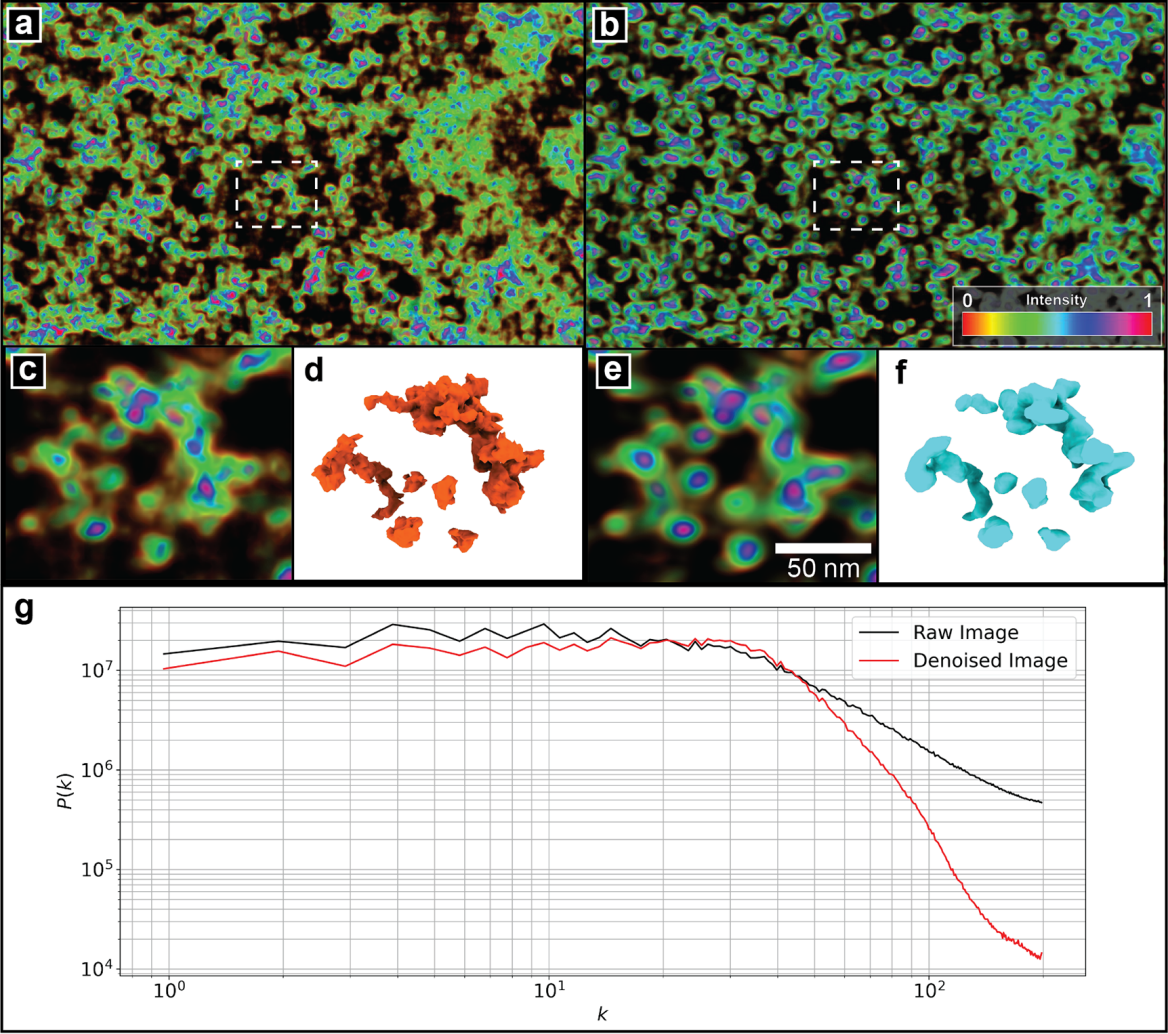
Application of the DAE to denoise the experimental tomogram of an imaged A549 cell. The (a) original experimental image and (b) image generated after passage through the trained DAE. To improve visual clarity and better highlight features of the images, the pixel intensities are normalized to a [0,1] scale and colored by a pseudocolor gradient indicated by the colorbar as opposed to a single grayscale channel. The denoised image achieves improved resolution of nucleosome-level features within chromatin-rich regions of the experimental image. A subsection comparison between the original (c) and denoised experiment (e) shows the reduction of noise and results in a smoother 3D reconstruction of the chromatin fiber from the denoised image (f) compared to the original (d). (g) A comparison of the power spectral density (PSD), *P*(*k*), between the raw and denoised images shows the denoised image to preserve the large-scale, low-frequency energy density at small wavenumbers *k* corresponding to the morphological structure of the chromatin fiber and attenuate the small-scale, high-frequency components at high *k* that can be primarily attributed to noise.

After validating that our DAE was capable of removing noise while preserving local structural features from our synthetic data set, we move to apply it to experimental ChromSTEM images of chromatin. Figure 5 shows the difference between a raw and denoised experimental tomogram of an imaged human pulmonary adenocarcinoma epithelial cell (A549 cell). A pseudocolor gradient as opposed to a single grayscale channel is employed to display pixel intensity for better visibility and to more clearly highlight the features within the image. Visual inspection of the denoised experiment confirms the ability of our DAE to remove noise and its ability to better resolve nucleosomes within chromatin-dense regions. Closer inspection of a randomly selected region of the denoised image (Figure 5b,e,f) clearly reveals the existence of clusters of a few nucleosomes that previous studies have suggested may play a role in the formation of topologically associated domains (TADs) in chromatin biology and which are much less clearly resolved in the original image (Figure 5a,c,d).^10^ We also compare the power spectral densities (PSDs) of the raw and denoised image stacks (Figure 5g). We see good agreement of the PSD at lower wavenumbers, which correspond to the large-scale (i.e., low-frequency) structural and morphological features of the image. At higher wavenumbers, the PSD of the denoised image exhibits a linear decrease relative to the raw image, which can be interpreted as the attenuation of small-scale (i.e., high-frequency) noise in the experimental image. Taken together, these results indicate that the important structural signal within the experimental ChromSTEM image is preserved by our denoising approach and produces superior resolution of nucleosome-level features within the chromatin-rich regions of the image.

**Figure 6:**
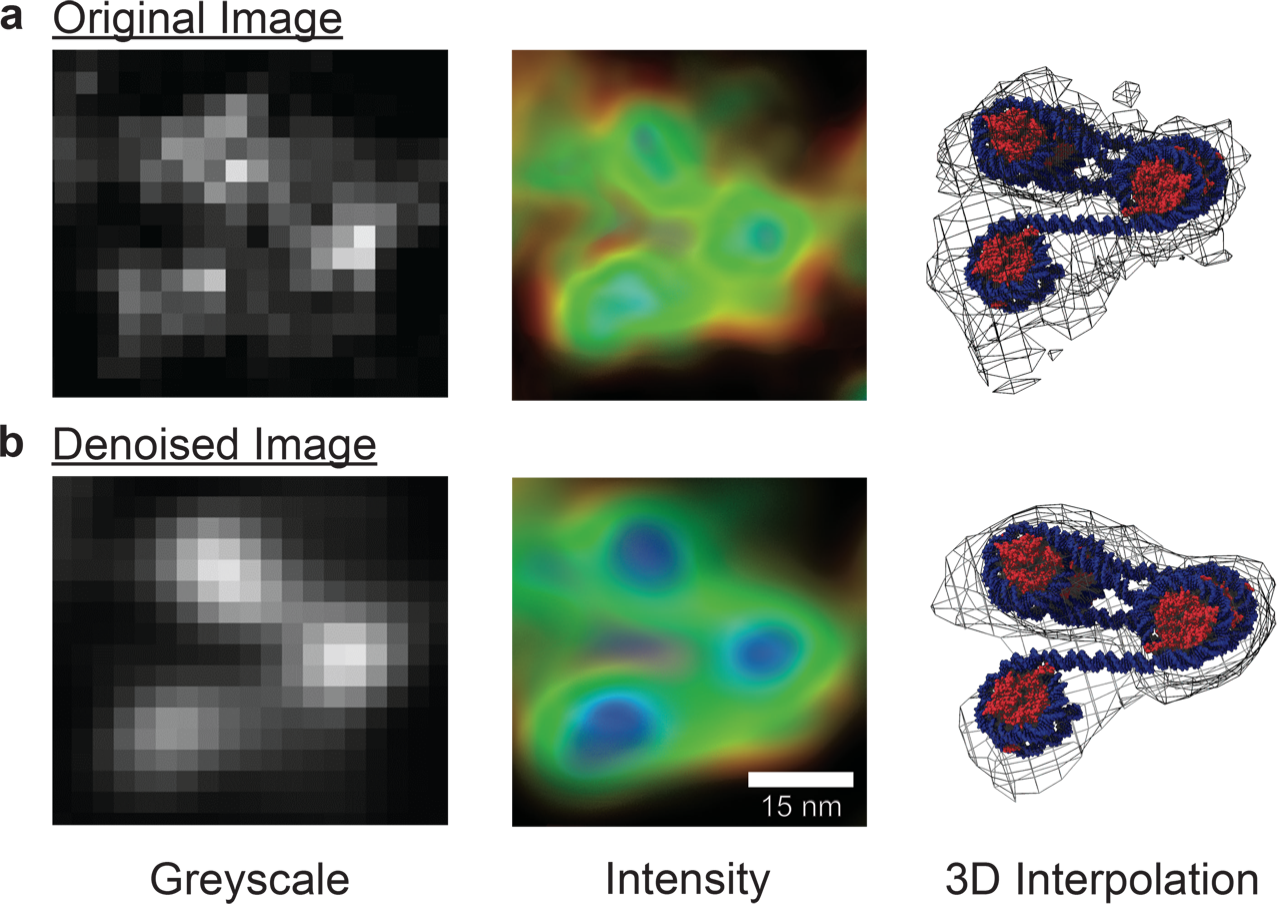
Denoised ChromSTEM images reveal tetranucleosome motifs within a dense chromatin cluster. Analysis of nucleosome clusters extracted from chromatin-rich regions of the (a) raw experimental tomogram and (b) after passing through the DAE. The denoised image clearly shows the presence of α-tetrahedron motifs that are difficult to discern in the raw image. Using Chimera, we construct a prototypical tetranucleosome motif (PDB: 1KX5) within the extracted volume of our denoised tomogram and find an optimal fit with an average high correlation score of 0.87.^56^ The construction of the 3D interpolation from the 2D imaging slices is computationally expensive but can, in principle, be extended to large sections of chromatin using high-performance computing resources.

**Figure 7:**
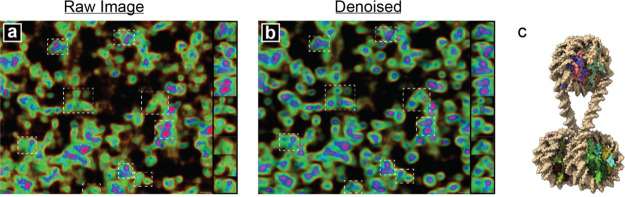
Denoised ChromSTEM images reveal tetranucleosomes motifs within dense chromatin clusters. Analysis of nucleosome clusters extracted from chromatin-rich regions within a 200 × 200 nm^2^ section of the (a) raw experimental tomogram and (b) after passing through the DAE. The denoised image clearly shows the presence of (c) α-tetrahedron motifs that are difficult to discern in the raw image. We find no evidence for β-rhombus motifs or for the 30 nm fiber.

To determine whether these small nucleosomal clusters are comprised of either of the two recently identified folding motifs (α-tetrahedron or β-rhoumbus), we visually inspect a number of nucleosome clusters extracted from chromatin-rich regions within a 50 × 50 nm section of the experimental tomogram (Figure 6). It is challenging to discern from inspection of the raw image, but after passage through the DAE it is visually apparent that these chromatin-dense regions are primarily composed of tetranucleosome motifs (Figure 7). To quantify our assertion, we construct a density map from our denoised STEM image stack and fit a prototypical α-tetrahedral tetranucleosome folding motif reconstructed from a single atomic nucleosome structure (PDB: 1KX5).^23^ To find an optimal fit, the cross-correlation coefficient (CCC) score was used to maximize the fit of a simulated map from the atomic structure and our volume map using the density mapping algorithm from the Chimera software.^56^ We find an improved optimal fit with an average high correlation score of 0.87 versus a correlation score of 0.85 for the original tomogram (Figure 6). Though comparatively small, incremental quantitative improvements can provide insightful details about the chromatin structure. Detecting and quantifying tetranucleosome motifs in raw and denoised images remains an important task and a significant challenge in the field, and expert experimentalists are crucial for interpreting results due to their deep understanding of the biological context and ability to assess image quality and identify relevant features.^57,58^ Our denoising method improved the detection of tetranucleosome motifs primarily based on visual cues, resulting in a more accurate representation of chromatin structure in denoised chromSTEM images (Figure 7).

These tetranucleosome motifs are known to promote DNA compaction and lead to chromatin condensation, and the preponderance of these structural elements observed within chromatin-dense regions is consistent with prior experiment and simulation.^10,11^ In contrast, we do not observe any zigzag β-rhombus motifs or find any evidence for the formation of the postulated 30 nm fiber.^59^ These results support a model in which the *in situ* structural organization of chromatin within chromatin-dense regions in the cell is not a 30 nm fiber, but rather largely composed of smaller tetranucleosome motifs.

### Identifying Packing Domains and Their Statistical Properties From Denoised ChromSTEM Stack

The denoised images produced by the DAE enable more robust resolution of chromatin-rich packing domains and improved estimation of statistical distribution of their structural properties such as size, packing scaling exponents, and chromatin volume concentration. We first describe these analyses in the context of the raw ChromSTEM images and then demonstrate how our statistical resolution improves within the denoised images.

Considering first the raw 3D ChromSTEM tomogram presented in Figure 5a, we extracted 76 chromatin-rich packing domains and then subjected them to structural analysis to determine the distribution of domain sizes *R*_*f*_. To do so, we adopted two complementary definitions of domain size. First, we identified the centroid of each domain by creating a local chromatin intensity map by applying Gaussian filtering and local contrast enhancement to the grayscale ChromSTEM z-stacks. We appeal to the fact that ChromSTEM intensity is approximately linearly proportional to mass to fit a scaling law between mass *M* and distance *r* from the centroid of each domain.^15^ Following classical power-law polymer scaling relations, mass and distance are expected to be related as *M*(*r*) ∝ *r*^*D*^, where *M* is defined as the integrated mass (i.e., intensity) lying at a particular radial distance *r* from the domain centroid and *D* is the packing scaling exponent for the polymer that is anticipated to be approximately constant over a particular range of length scales.^60^ We computed best-fit values of the packing scaling exponent *D* by fitting power laws over the range of [0,*r*] at increasing *r* and defined the domain size *R*_*f*_^(1)^ as the distance *r* at which we observe more than 5% deviation from the best-fit power law. This demarcates the length scale at which a single power-law relationship no longer holds and constitutes our first definition of *R*_*f*_ (Figure S1a,b). Second, we calculated the radial density profile of chromatin as a function of distance *r* from the centroid of the domain. This profile is expected to monotonically decrease until the distance *r* reaches the boundary of the domain and then increase again as it begins to encroach upon a neighboring domain (Figure S1c). The minimum in the radial density profile defines our second definition of domain size *R*_*f*_^(2)^. Finally, we defined the domain size *R*_*f*_ = min(*R*_*f*_^(1)^, *R*_*f*_^(2)^). We observe that the two complementary definitions of domain size over which we take the minimum are necessary to properly account for the environment in which the domains may be found: in chromatin-poor environments where the domains are isolated, we expect domain-size to be dictated by the mass distribution of the single domain under consideration and *R*_*f*_^(1)^ < *R*_*f*_^(2)^; in chromatin-rich environments, we anticipate *R*_*f*_^(2)^ < *R*_*f*_^(1)^ and domain size should be more appropriately defined as an multibody property that defines the boundary between domains.

Having defined *R*_*f*_ and *D* for each domain, we compute the chromatin volume concentration, CVC, which correlates with the binding efficiency of transcriptional reactants and is defined as the fraction of volume occupied by chromatin.^13,15^ The CVC was calculated as the total number of nonzero voxels over the total number of voxels per domain.^15^ The distributions of these three quantities for the 76 chromatin-rich domains extracted from the raw A549 3D ChromSTEM tomograms are presented in Figure S2, for which we report means and standard deviations of *R*_*f*_ = 71 ± 26 nm, *D* = 2.46 ± 0.18, and CVC = 42 ± 14%. We previously demonstrated that chromatin forms spatially well-defined higher-order domain structures with radii ranging between an interquartile range of 60–90 nm in A549 cells and observe that our present measure of mean domain size lies squarely within this range.^15^

**Figure 8:**
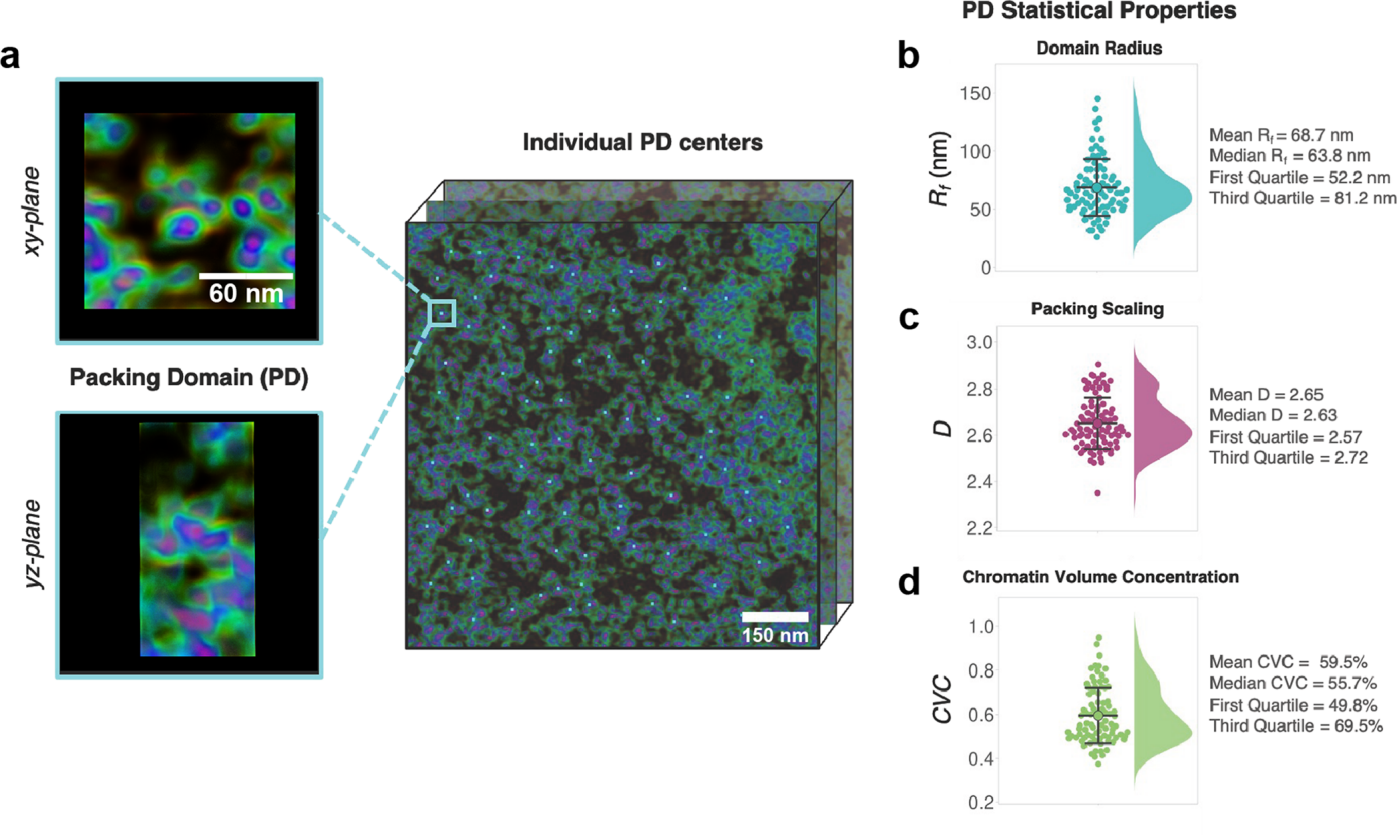
Structural analysis of chromatin-rich packing domains from the DAE-denoised A549 3D ChromSTEM tomogram. (a) A 3D conformation of a packing domain identified from the denoised ChromSTEM tomogram (Figure 5b). Statistical distribution of (b) domain size *R*_*f*_, (c) packing scaling exponent *D*, and (d) cluster volume concentration CVC, over the 85 chromatin-rich packing domains identified from the denoised ChromSTEM tomogram. Denoising enables identification of ∼12% more domains and domains more closely associated in space relative to analysis of the raw 3D ChromSTEM tomograms.

A concern of applying this structural analysis to the raw ChromSTEM tomograms is the introduction of errors into both the definition of the domains and their structural properties due to the noise inherent in the experimental images. Accordingly, we repeated this analysis for the DAE denoised 3D ChromSTEM tomogram presented in Figure 5b. In doing so, our procedure identified 85 chromatin-rich packing domains, 9 more than were identified in the raw images. An analysis reveals that application of the domain identification procedure to the denoised image enables identification of more domains and better resolves domains more closely packed in space (Figure S3). The improvement in signal-to-noise ratio in the denoised tomogram appears to assist in the identification of domain centers that cannot be resolved in the raw tomogram and which are confirmed by manual visual analysis. To assess the possibility of introducing artifacts through the DAE denoising, we present in Figure 8 the statistical analysis of *R*_*f*_, *D*, and CVC over the 85 denoised ChromSTEM domains. The mean reported values of *R*_*f*_ = 69 ± 24 nm, *D* = 2.65 ± 0.11, and CVC = 60 ± 13% are all in good agreement with the analysis of both the raw ChromSTEM images and our prior analyses^15^ but are now based on better statistics enabled by the identification of ∼12% more domains in the denoised images.

## Conclusions

By leveraging molecular dynamics and machine-learning approaches, we constructed and trained a denoising autoencoder (DAE) capable of removing noise commonly found in scanning transmission electron microscopy tomography with ChromEM staining (ChromSTEM) imaging. The model is trained over physics-based coarse-grained molecular dynamics simulations using the 1CPN model and learns to distinguish the signal from ground truth chromatin structures from artificial noise mimicking the noise profile inherent to experimental STEM imaging. In tests on synthetic ChromSTEM images generated by molecular simulations for which the ground truth is exactly known, the training outperforms standard denoising approaches, offering a 57% improvement in the mean squared error relative to block-matching and 3D filtering and a 72% improvement over nonlocal means. In applications to *in situ* experimental ChromSTEM images of chromatin within human pulmonary adenocarcinoma epithelial cells (A549 cells), we demonstrate that the DAE eliminates high-frequency noise while preserving the large-scale signal characterizing the chromatin organizational structure. The denoised images enable identification of tetranucleosome motifs at a resolution inaccessible within the raw images and expose the α-tetrahedron as the predominant organizational subunit within chromatin-dense regions in the cell and which have been suggested to play a role in chromatin compaction and regulation of gene expression. Notably, we find no evidence for the presence of β-rhombus tetranucleosome motifs or for the 30 nm fiber. The denoised images also permit the identification of ∼12% more chromatin-rich packing domains that are obscured by noise within the raw images, enabling improved statistical resolution of the distribution of domain sizes, packing scaling exponents, and chromatin volume concentrations without apparently introducing statistical artifacts. The domain size distributions are consistent with, but have higher statistical resolution and smaller uncertainties than, our prior analyses.^15^

The nucleosome motifs exposed by this approach enable a new understanding and insight into the small-scale structural organization of chromatin within the cell and how these structures can influence DNA accessibility and gene regulation. The present work focused primarily on the analysis of tetranucleosome motifs, but in future work we hope to expand our focus to smaller di- and trinucleotide motifs. We anticipate that the approaches reported in this study may be applied to ChromSTEM imaging to advance our understanding of how stress and epigenetic factors affect chromatin conformation and gene regulation and may also be applied to other imaging techniques such as cryogenic electron microscopy (cryo-EM). Our study also exemplifies a generic paradigm wherein experimental imaging and theoretical modeling may be bridged via machine-learning approaches to enable high-resolution exploration of structural organization within biological systems.

## Supporting Information Available

Mass scaling and radial chromatin density of chromatin domains, morphology of A549 cells characterized in a noisy tomograph, and denoised tomographs revealing additional domains (PDF)
