# Supplementary material for: Denoising Autoencoder Trained on Simulation-Derived Structures for Noise Reduction in Chromatin Scanning Transmission Electron Microscopy
Source: ACS Cent Sci. 2023 Jun 5;9(6):1200–12. doi: 10.1021/acscentsci.3c00178 (PMC10311656; doi:10.1021/acscentsci.3c00178)
Supplement: Supplementary file 1 — oc3c00178_si_001.pdf [file oc3c00178_si_001.pdf]

SUPPORTING INFORMATION: Denoising Autoencoder Trained on Simulation-Derived Structures for Noise Reduction in Chromatin Scanning Transmission Electron Microscopy

Walter Alvarado,[†] Vasundhara Agrawal,[‡] Wing Shun Li,[¶] Vinayak P. Dravid,[§] Vadim Backman,[*,‡,¶] Juan J. de Pablo,[*,‖] and Andrew L. Ferguson[*,‖]

†*Biophysical Sciences, University of Chicago, Chicago, IL 60637, USA*

‡*Department of Biomedical Engineering, Northwestern University, Evanston, IL 60208, USA*

¶*Department of Applied Physics, Northwestern University, Evanston, IL 60208, USA*

§*Department of Materials Sciences and Engineering, Northwestern University, Evanston, IL 60208, USA*

‖*Pritzker School of Molecular Engineering, University of Chicago, Chicago, IL 60637, USA*

E-mail: v-backman@northwestern.edu; depablo@uchicago.edu; andrewferguson@uchicago.edu

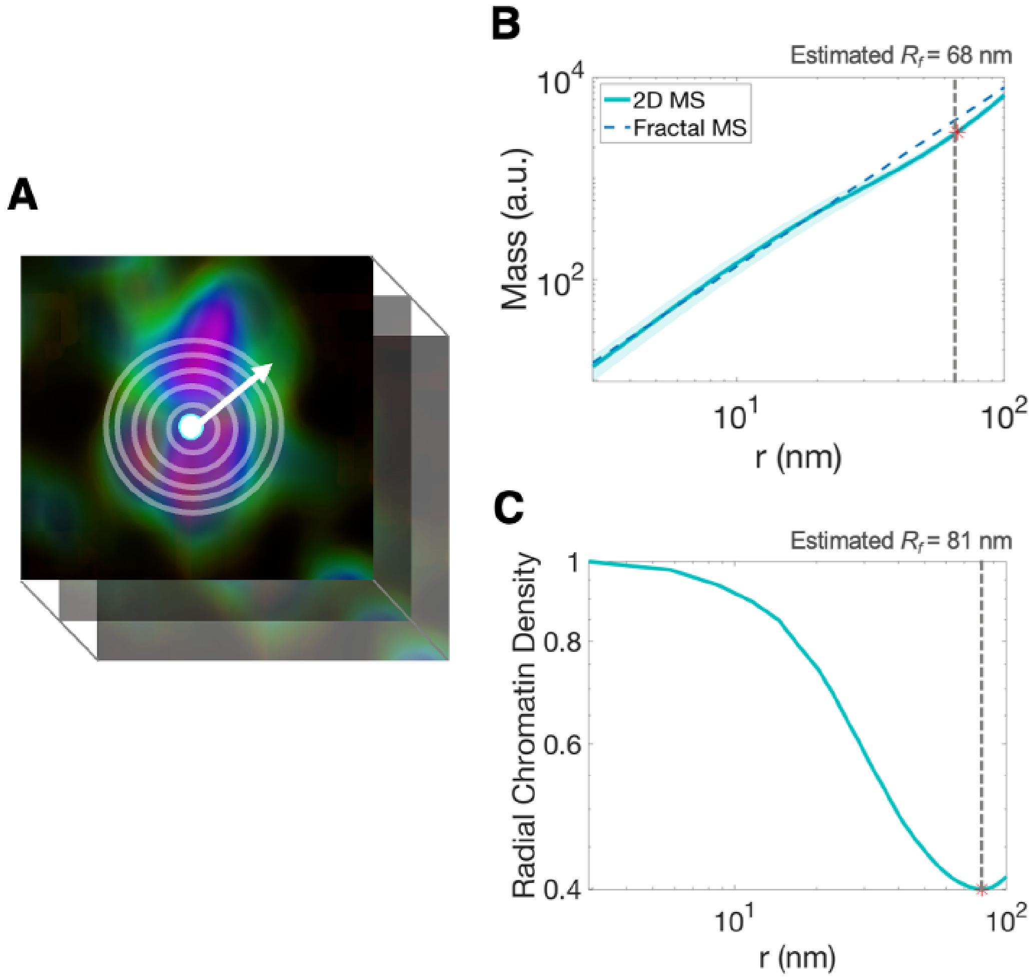

Figure S1: Mass scaling and density analysis originating from the domain centers. A) Mass and radial chromatin density are evaluated starting from the center of a domain (white circle with cyan outline) in concentric circles with increasing distance, r. B) Mass scaling of an individual domain in the log-log scale. We performed linear regression on the mass scaling curve and obtained a slope, $D < 3$ for r up to 68 nm (blue dashed line). Beyond the red asterisk, a more significant divergence (>5% error) in the mass scaling behavior is observed. Further, as r increases, there is a sharp transition to the supra-domain regime with D approaching 3. C) Radial chromatin density of an individual domain in the log-log scale. Radial chromatin density of a domain initially is almost constantly high, roughly near the center of the domain. The density then decreases rapidly at moderate distances from the domain center. After a given large distance shown as a red asterisk at 81 nm, radial density increases again. This increase is potentially due to the end of one domain boundary and the interactions with neighboring domains.

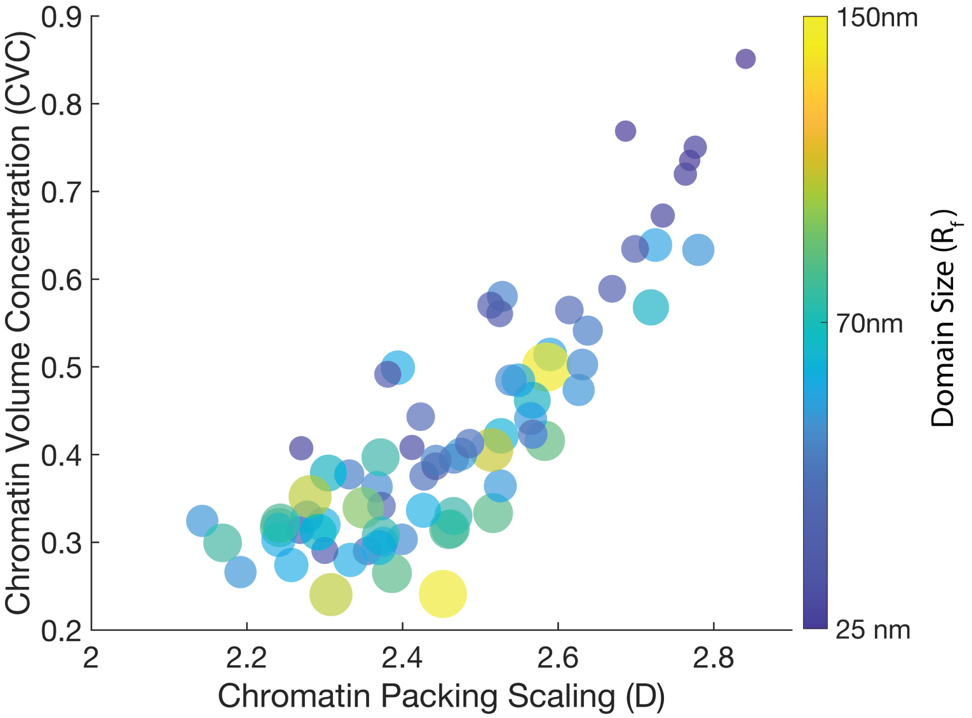

Figure S2: Characterization of morphological properties of original higher-noise tomograph of A549 cells. Statistical distribution of chromatin packing scaling D, cluster volume concentration CVC, and domain size R_f.

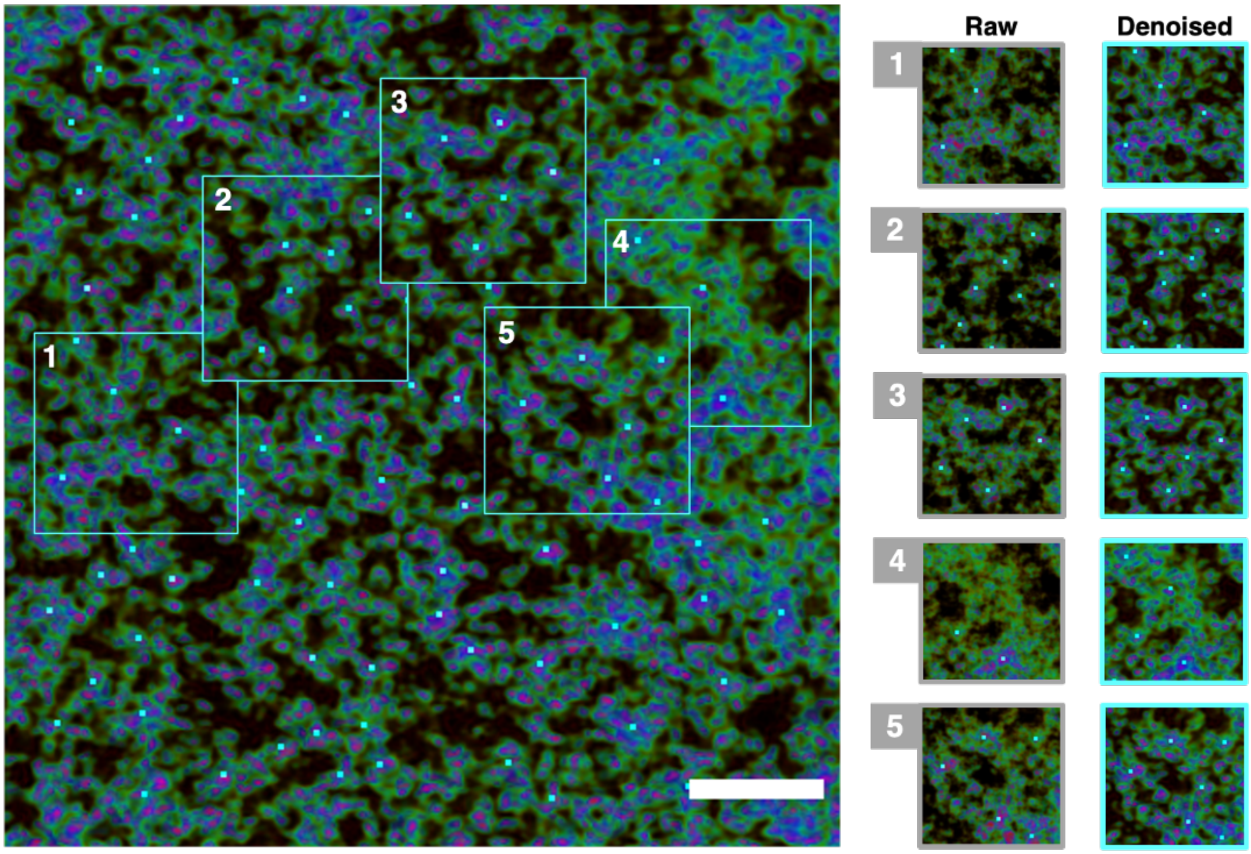

Figure S3: Denoising can resolve domains that are closer in space. Left: Domain centers were estimated from denoised tomograms. Right: Five representative regions of the raw and the denoised tomograms show that more domains were identified in the denoised tomogram. Centers are indicated in cyan.
